# A Health Technician-delivered Brief Intervention linked to AUDIT for reduction of alcohol use in Chilean primary care: a randomized controlled trial

**DOI:** 10.1186/s13722-021-00248-4

**Published:** 2021-06-15

**Authors:** Nicolas A. Barticevic, Fernando Poblete, Soledad M. Zuzulich, Victoria Rodriguez, Diego Quevedo, Brena F. Sena, Laura Bradshaw

**Affiliations:** 1 School of Medicine, Department of Family Medicine, Pontificia Universidad Católica de Chile, Alameda 340, 8331150 Santiago, Chile; 2 School of Medicine, Department of Public Health, Pontificia Universidad Católica de Chile, Alameda 340, 8331150 Santiago, Chile; 3 Pontificia Universidad Católica de Chile, Nursing School. Alameda 340, 8331150 Santiago, Chile; 4 Department of Epidemiology, Harvard T.H. Chan School of Public Health, 677 Huntington Ave, Boston, MA 02115 USA

**Keywords:** Alcohol brief intervention, Chile, Primary care, Health technician

## Abstract

**Background:**

Because of the shortage of health professionals in Chilean primary care, Health Technicians (HT) are providing Brief Interventions (BI) for risky alcohol consumption. We compared the efficacy of two AUDIT-linked interventions provided by HTs: an informative leaflet and a BI plus leaflet.

**Methods:**

This is a parallel-group randomized controlled trial with 1:1 randomization. Participants were identified through screening with the Alcohol Use Disorders Identification Test (AUDIT) at five primary care centers between March 2016 and July 2017. People older than 18 years at intermediate-risk (AUDIT score 8 to 15, inclusive) were randomized to receive either an HT-delivered BI (n = 174) or an informative leaflet (n = 168). Only data from participants (n = 294) who completed the 6-month assessment were analyzed. The leaflet was delivered without further advice. It contains alcohol consumption limits, a change planner, and strategies to decrease drinking. The BI was a 5-min discussion on the leaflet´s content plus normative feedback, tailored information on alcohol and health, and a change plan. The change in the AUDIT risk category six months after randomization (primary outcome) was compared among groups with a Chi-squared test. Changes in the secondary outcomes, which were scores on the AUDIT and the AUDIT´s consumption items (AUDIT-C), were compared with T-tests. Mixed-effects linear models adjusted for potential confounders. Outcome adjudicators were blinded to group assignment.

**Results:**

At 6-month follow-up, low-risk alcohol consumption was observed in 119 (80%) participants in the BI group, and in 103 (71%) in the leaflet group, with no difference among groups ($\chi 2$ [1, N = 294] = 2.6, p = 0.1; adjusted odds ratio 0.6; 95% confidence interval [CI] 0.34, 1.05). The mean AUDIT score decreased by 5.76 points in the BI group, and by 5.07 in the leaflet group, which represents a 0.86 AUDIT point reduction attributable to the BI (secondary outcome) (T = 2.03, p = 0.043; adjusted mean difference 0.86 CI 0.06, 1.66).

**Conclusions:**

The AUDIT-linked BI delivered by HTs was not associated with a greater reduction of risky alcohol consumption than an informative leaflet. Delivering a leaflet could be more efficient than a BI when provided by HTs; however, more research on the effectiveness of the leaflet is needed.

*Trial registration* ClinicalTrials.gov NCT02642757 (December 30, 2015) https://clinicaltrials.gov/ct2/show/NCT02642757.

## Background

Worldwide, alcohol consumption is the cause of 5.1% of years of life lost due to disability (DALYs) [1]. In Chile, the situation is even worse, with 12% of DALYs being caused by alcohol consumption and alcohol misuse, surpassing factors such as obesity, hypertension, and tobacco use [2]. Considering this alarming health burden, policies and strategies to address this issue have been progressively implemented within Chile’s public health system [3], including the National Screening, Brief Intervention (BI) and Referral to Treatment (SBIRT) program in primary care [4]. This strategy strives to fulfill two purposes in the Chilean health system: as stand-alone interventions for people with low and medium levels of alcohol use, and as the first intervention for people requiring referral to secondary care to treat their alcohol use disorder.

The SBIRT program was created in 2011 and has been implemented in the primary care network of the public health care sector [5], which serves around 80% of the Chilean population. In line with recommendations from international agencies such as the World Health Organization [6], Chile’s Ministry of Health (MOH) has promoted the implementation of the SBIRT program to reduce the social and health consequences resulting from excessive alcohol use. In 2018, 1,268,640 people were screened, and 75,854 received a BI in the public health care sector [7], which represents a population coverage of approximately 8% [8]. These figures do not include the services provided in the private health care sector, where the SBIRT program has not yet been widely adopted. Altogether, access to health services for people with harmful or problematic alcohol use is insufficient in Chile, and this is also the case for over 70% of Latin America countries that report limited or scarce access to health services for alcohol [1].

In the SBIRT program’s first stage of implementation, the BIs were administered in primary care centers exclusively by traditional health professionals, such as doctors, nurses, and midwives. However, this strategy provided insufficient coverage due to the shortage in the number of these health professionals and their burden of other critical tasks [9]. To overcome this barrier, many health centers turned to health technicians (HTs) for assistance and extended support in delivering BIs.

The delivery of BIs by other members of the health team is of much interest in health systems where there are shortages of traditional medical professionals. In Chile, primary care centers are composed of a multidisciplinary team, including physicians, nurses, social workers, psychologists, dentists, kinesiologists, and Health Technicians (HTs), among others [10]. As part of the team, HTs currently undertake routine health tasks, including measuring patients´ vital signs before a doctor or nursing visit and performing much of the patients´ annual check-up. Therefore, HTs are in a natural position to provide alcohol use screenings and BIs.

Despite the potential for incorporating HTs into the SBIRT program, the efficacy of an HT-delivered BI has not been tested. When Chilean health centers incorporated these providers into the SBIRT program, they assumed that the effectiveness of the HT-delivered BI was similar to a health professional-delivered BI. However, there are some reasons to question this equivalence. For example, although an HT degree in Chile involves two and a half years of education and training, it does not include advanced training in clinical interview skills, which may affect the quality of the BIs provided. Besides, in Chilean culture, HTs do not have the same level of influence on patients as do doctors and nurses [11]; thus, an HT-delivered intervention could be less efficacious [12].

Moreover, the rationale for disseminating SBIRT programs in Chile rests mostly on the substantial evidence of the effectiveness of BIs in studies with physicians and nurses, mainly in developed European and North American countries [13]. Currently, only one qualitative report has been published on the barriers and facilitators in implementing BIs by HTs in the Chilean health care system [14]. Thus, it is essential to evaluate the efficacy of the HT-delivered BI within the SBIRT program and the local Chilean context.

This randomized controlled trial (RCT) represents the first step to evaluate the efficacy of a BI linked to the Alcohol Use Disorders Identification Test (AUDIT) provided by HTs in primary care in Chile [15], and, as far as we know, in Latin America. We compared the efficacy of two interventions provided by HTs immediately after completing the AUDIT: an informative leaflet and a BI plus leaflet, in five primary care centers in Chile.

We hypothesized that the AUDIT-linked BI delivered by HTs would lead to greater reductions in risky alcohol use in comparison to an informative leaflet. We chose the leaflet as a comparison because it represents the minimal level of intervention within the Chilean SBIRT program; therefore, the comparison will provide evidence on the efficacy of an HT-delivered BI relevant to the local context of SBIRT program delivery. We believe this study contributes to understanding the efficacy of an alcohol BI when delivered by HTs in primary care. Also, our results can be relevant to countries with similar primary care services, where HTs and other health educators play an essential role.

## Methods

### Overview and design

This study is an open RCT, of parallel groups, with 1:1 randomization ratio. Participants were randomly assigned to receive an informative leaflet or a BI plus leaflet linked to the AUDIT, and were re-evaluated six months later.

Although we studied the performance of the BI in near real-world conditions (i.e., delivered by HTs within primary care), this study is best described as an efficacy trial since it departs from real-world conditions in several ways [16]. While the HTs were current workers at the participating health centers, they received specific training, provided by the research team, and only the HTs that demonstrated proficient delivery of the BI and protocol adherence were selected. As we planned to conduct the study in close to real-world conditions, we anticipated that the HTs would conduct the research procedures during their daily practice. However, the study's pilot showed that it was unfeasible to adapt their daily routines to accommodate the research procedures. Therefore, we decided to directly hire the participating HTs to work part-time so that they could have sufficient time to dedicate to the study. On average, about half of their time was paid by the study. In their remaining work hours, the HTs continued working in the health centers carrying out their regular, non-study related tasks.

This study was funded by FONIS (National Health Research Fund) and executed in collaboration with the municipalities of San Miguel, Puente Alto, and La Pintana in Santiago, Chile, between March 2016 and July 2017. Participants were recruited from five primary health centers located within those municipalities: San Alberto Hurtado (n = 117), Madre Teresa de Calcutta (n = 40), Recreo (n = 10), Barros Luco (n = 70) and Juan Pablo II (n = 105). These centers are representative of a typical Chilean primary care center in the public health system; each serves a potential population between 20,000–30,000 individuals and provides a wide arrange of services, such as immunizations, social services, and several professional consultations (e.g., psychologist, midwives, dentist, physician). The study protocol was approved by the Scientific Ethics Committee of the School of Medicine of the Pontificia Universidad Católica de Chile, as well as the Scientific Ethics Committees of the South East Metropolitan Health Service and the Central Metropolitan Health Service, which correspond to the participating municipalities.

### HTs training and selection

This process consisted of two sequential phases: training and selection. First, 58 HTs from the five centers participated in two 4-h training sessions dedicated to the theoretical and practical aspects of BI, provided by the researchers (SZ, VR, and NB). Here, they reviewed the administration and scoring of the AUDIT and the rationale behind the SBIRT program strategy, and received a primer on the spirit of the Motivational Interview (MI) [17]. The sessions ended with a demonstration of a well-conducted BI by one of the researchers and with multiple role-playing exercises.

After the training was completed, HTs participated in a simulated participant session where the HTs provided the AUDIT and BI. The researchers (SZ, VR, and NB) selected HTs that demonstrated proficiency with each of the BI components, and notably, those who could provide non-judgmental feedback and maintain the MI spirit throughout the practice intervention. An ad-hoc observation form (see Annex, part 5) was constructed to assist in the selection. We aimed to include all the HTs that are part of the SBIRT program in each center and that achieved BI proficiency after participating in the training. From the 58 HTs that received training, 32 HTs were selected, but only ten participated in the study due to administrative and practical constraints. These ten HTs received additional training in the study protocol procedures (obtaining consent, randomization procedure, and record-keeping).

### Recruitment and baseline study procedures

Participants were identified during their preventive annual health assessment, which routinely includes the AUDIT. The HTs provided only the alcohol use component of the check-ups; that is, they verbally administered the AUDIT and used the AUDIT score to screen for potential participants. Patients aged 18 years or older with AUDIT scores between 8 and 15 (i.e., intermediate-risk) were invited to participate by the same HT. The aim of the study and its procedures, including the study randomization, were explained to each potential participant. Upon obtaining written informed consent, the HT opened an opaque envelope containing the intervention allocation and then performed the corresponding intervention. Patients who did not consent to participate received the BI without being randomized nor included in the study. For practical and ethical reasons, it was not possible to blind the participants to the purpose of the study or to the allocated intervention group.

We excluded individuals that were in treatment for alcohol use disorder or who specifically sought help for this; did not speak Spanish; and pregnant women, who are referred directly to medical evaluation as established by national technical standards. Individuals with problematic use of alcohol (i.e., AUDIT score higher than 15 points) were not eligible for the study, but did receive the BI and were referred for further medical evaluation in the same health center.

### Description of the intervention conditions

a. Leaflet: in this group, the HTs informed the participant that their alcohol use was risky, handled them the leaflet [18], and invited them to read it at home. This interaction lasted for less than a minute and did not involve any further explanation or advice. The leaflet contains (a) information on national data on alcohol consumption; (b) the maximum alcohol limit per occasion (i.e., three standard drinks for women and four for men) and the maximum weekly limit (i.e., seven standard drinks for women and fourteen for men); (c) a daily and weekly goal setting planner; (d) three specific strategies to decrease alcohol use (i.e., have a maximum of one drink every 90 min, eat food along with alcohol, and drink non-alcoholic drinks in between drinks containing alcohol); (e) and the warning to never drink while driving or while pregnant.

b. Brief Intervention: in this group, the HTs first invited the participants to review the results of the AUDIT, informed them of their risk status, and then provided normative feedback based on the content of the leaflet. The HTs gave them additional information regarding the effects of alcohol on at least one specific health topic that seemed relevant to the participant (e.g., the effects of alcohol on blood pressure or mental health). Then, they informed the participant about the alcohol use limits using part (b) of the leaflet and gave a non-judgemental but firm recommendation to suspend or reduce alcohol use. Participants were invited to set goals, and when they agreed, part (c) of the leaflet was used for this purpose. The intervention concluded with the discussion of leaflet's parts (d) and (e) and with the invitation to come back to the center for additional information or help if needed.

### Fidelity to the BI

The HTs were monitored every two weeks throughout the trial to assure fidelity to the BI and protocol integrity. A research assistant (RA) with training in MI and the BI model visited each center every two weeks and observed at least one BI and one leaflet delivery per HT. This supervision was assisted by a field observation form (Annex) that combined standards for protocol integrity and intervention fidelity, and yielded three possible outcomes: total, sufficient, or insufficient compliance.

The HTs that demonstrated total compliance could continue recruiting participants. The HTs that showed sufficient compliance were given feedback and recommendations for improvement and suspended recruiting until a new visit confirmed proficiency. None of the HTs showed insufficient compliance, which would have resulted in suspending their participation in order to be retrained.

### Instrument and outcomes

All outcomes were pre-specified in the trial protocol, which is available in Spanish [19]. The AUDIT validated in Chile [20] was used to measure all of alcohol outcomes. Its scores are equal to the original version.

The primary outcome measure of the study was the change in the AUDIT risk category six months after receiving the BI. We also measured the change in AUDIT total score and AUDIT-C score [21] after six months (i.e., the first three questions of the AUDIT instrument, which describe the quantity and frequency of alcohol use).

At entry, we recorded participants´ birthdate, sex, marital status, educational level, and employment. To better characterize the sample, we asked participants whether a physician had prescribed them any medication for mental health problems in the last year.

We did not measure other variables that could have been of interest, such as other drug use (including tobacco) or mental health symptoms. The main reason for this was to have research procedures that would not interrupt routine care and to promote patient participation.

Participants were contacted by Research Assistants (RAs) via telephone six months after receiving the BI or the leaflet, and were invited to a re-evaluation visit at the health center. When phone contact could not be established, a letter was sent to the participant, inviting them to visit the health center. The follow-up AUDIT was administrated by RAs blinded to the participant´s allocation. All measurements were performed in person. Upon completing the follow-up visit, participants received CLP $10.000 (USD $15) in compensation for their time. RAs were asked to not inquire about which group participants belonged to or what elements of the intervention they remembered in order to keep the masking intact and avoid confirmation bias.

### Sample size

A previous meta-analysis estimated a BI effect size of 0.14 at three to six months for drinking-related outcomes [22]. Consequently, we calculated that 109 participants were needed per group to detect a reduction of 14% in the risk status associated with the BI with a power of 80% and a type one error less than 5%. Additionally, we assumed a 20% loss to follow-up, so we aimed to recruit 262 participants.

### Random sequence generation

Randomization was generated by a member of the research team (FP) using the SAS software program for Windows (SAS Institute Inc., Cary, North Carolina, USA) with a 1:1 randomization ratio using blocks of 50 participants, and was distributed to the health centers in boxes with sealed opaque envelopes numbered consecutively. To minimize the risk of the randomization sequence being altered, the status of the envelopes was reviewed monthly by an RA. In the health centers, a trained HT recruited eligible participants and assigned them to receive the BI or the leaflet.

### Statistical analysis

The analysis of the outcomes included all participants who completed the follow-up appointment (n = 294). Participants were analyzed according to the group to which they were allocated. We did not attribute any missing data, under the assumption that loss to follow up was not associated with either a positive or a negative outcome.

For the primary outcome, a chi-squared test compared the AUDIT risk-category of the participants in each group at follow up. Then, a mixed-effects logistic regression was fitted to model the clustering of participants among health centers and to adjust for possible confounders (age, sex, and educational level). In this regression, the demographic variables were modelled as fixed effects, while the health center was regarded as a random effect variable to account for the variability within centers.

For the secondary outcomes, independent t-tests compared the AUDIT and AUDIT-C scores among groups at six-month follow-up. Then a mixed-effects linear model was used to adjust for sex, age, and educational level. Additionally, the model included the at-entry AUDIT score to adjust for regression to the mean. Similarly, in the logistic model the health center was included as a random variable.

The R statistical environment was used for the analyses [23], and particularly, the lme4 package for the mixed-effects models [24]. The full data and analyses' scripts are available through the Open Science Foundation repository [25].

## Results

### Characteristics of the participants

**Fig. 1:**
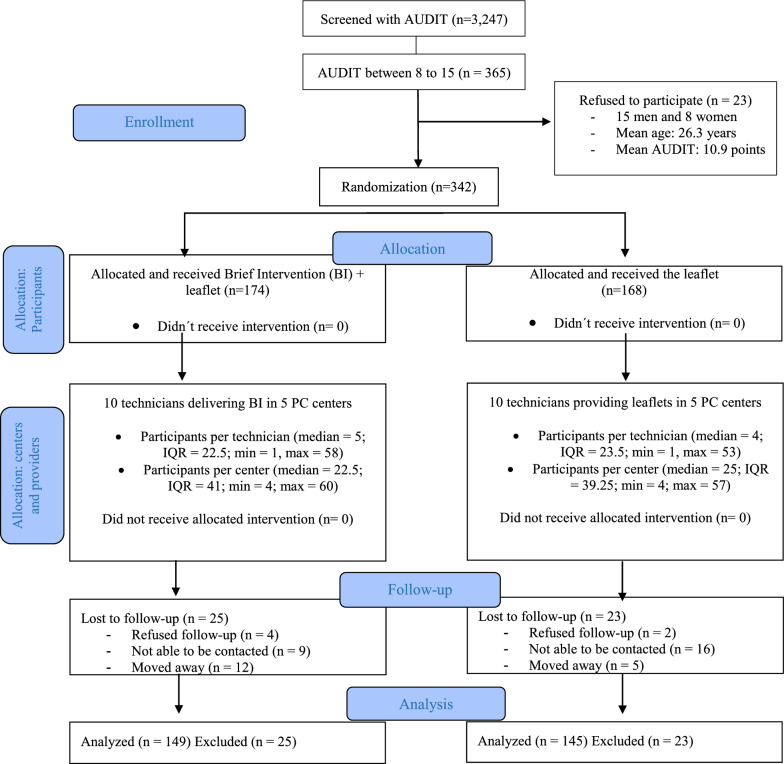
CONSORT diagram [44]

Of the 3,247 AUDITs applied during health check-ups between March 2016 and July 2017, 11% (357 patients) had intermediate-risk alcohol use on the AUDIT (scores 8 to 15). Of those, 342 patients agreed to participate and were randomized: 174 participants into the BI group and 168 into the leaflet group. Figure 1 shows the flow of the participants in the study.

The average age of study participants was 29 years old, and 57% were male. The average AUDIT score at entry was 10.5 (SD 2.2). There were no significant differences between the two groups in the demographic variables: age, sex, marital status, employment, educational level, mental health medications, or AUDIT score. Table 1 shows the demographic distribution of each group.

**Table 1:** Primary care users with intermediate-risk in the AUDIT, randomized to the study (n = 342)

	Brief Intervention + leaflet (n = 174)	Leaflet (n = 168)	p value
% Male (n)	55.7 (97)	58.3 (98)	0.71
Age, mean (SD)	28.8 (9.2)	29.5 (8.2)	0.49
% Married (n)	44 (25.3)	59 (33.9)	0.12
% Employed	65.5 (114)	60.3 (105)	0.28
Educational level % (n)			
Incomplete basic	9.7 (17)	14.3 (24)	0.73
Basic	62.1 (108)	57.8 (97)	
Technical or university	27.6 (48)	27.9 (47)	
Mental health medications in the last three months % (n)			
At-entry scores ($\mu$, SD)	14 (24)	21 (35)	0.1
AUDIT	10.4 (2)	10.6 (2.2)	0.4
AUDIT-C	6.1 (1.5)	5.9 (2)	0.51

$\mu , {\text{mean}}.$ SD, standard deviation. AUDIT: Alcohol Use Disorders Identification Test. AUDIT-C: three first items from the AUDIT

At six-months after randomization (184.2 days SD 39.6), 294 (86%) of the participants returned for the follow-up visit and completed a new in-person AUDIT. The follow-up rate was not significantly different between groups [$\chi$^2^ (1, N = 294) = 0.0006, p = 0.9], nor was the follow-up time (185.1 days in the BI group and 183.2 in the leaflet group, p = 0.67). Only the participants that completed the AUDIT at six months were analyzed for the outcomes, while those who did not complete the follow up assessment (n = 48) were excluded from the analyses. Participants lost to follow up were more likely to be men (OR 2.5, p < 0.01), but did not differ in any other at-entry characteristic.

### Outcomes

The AUDIT risk category (primary outcome) was compared between groups at follow-up (Table 2). A reduction from the intermediate to the low-risk category was observed in 119 (80%) of 149 participants randomized to the BI group and 103 (71%) of 145 participants randomized to the leaflet group, but this 9% difference did not reach statistical significance: [$\chi$^2^ (1, N = 294) = 2.6, p = 0.1]. In the multivariate analysis, the adjusted odds ratio was 0.6 [95% confidence interval (CI) (0.34, 1.05)].

**Table 2:** Primary outcome. Change in risk category in both groups at 6 months follow-up

	Maintained or increased risk	Lowered risk	Odds Ratio	Adjusted* Odds Ratio	Adjusted* confidence interval**	Adjusted* *p* value
Brief intervention + leaflet (n = 149)	30	119	0.62	0.60	[0.34, 1.05]	0.07
Leaflet (n = 145)	42	103				

Primary outcome : C hange in AUDIT risk category (0 – 7 low-risk, 8 – 15 intermediate-risk, 16 – 40 high risk)

^*^ Mixed-effects linear model that adjusted for age, sex, and educational level as fixed-effect predictors, and health center as a random-effect variable. ** 95% confidence interval

AUDIT: Alcohol Use Disorders Identification Test

Table 3 shows the comparison of the AUDIT and AUDIT-C scores among both groups. At the six-month follow-up, the average AUDIT score lowered from 10.4 to 4.64 in the BI group, and from 10.6 to 5.53 in the leaflet group, which represents a difference of 0.89 points in favor of the BI [t(290) = 2.03, p = 0.043].This difference was maintained when adjusting for the initial AUDIT score, sex, age, educational level, and health center [adjusted mean difference 0.86, CI (0.08, 1.69), p = 0.031]. Additionally, the AUDIT-C score was lower by 0.38 points in the BI group, but this difference was not significant in the mixed effects model [adjusted mean difference 0.44, CI (0, 0.88), p = 0.052].

**Table 3:** Secondary outcomes

	Baseline	Six months	Mean Difference (95% CI)	Adjusted Mean Difference (95% CI)*
AUDIT				
Brief intervention + leaflet (n = 149)	10.4	4.64	0.89 [0.03, 1.74]	0.86 [0.08, 1.69]
Leaflet (n = 145)	10.6	5.53		
AUDIT-C				
Brief intervention + leaflet (n = 149)	6.06	3.07	0.38 [− 0.07, 0.84]	0.44 [0, 0.88]
Leaflet (n = 145)	5.95	3.46		

AUDIT and AUDIT-C scores for both groups at admission and 6 months

Secondary outcomes : A UDIT total and AUDIT-C scores

^*^ Mixed-effects linear model that adjusted for entry AUDIT score, age, sex, and educational level as fixed-effect predictors, and health center as a random-effect variable

*AUDIT* Alcohol Use Disorders Identification Test, *AUDIT-C* three first items from the AUDIT, *SD* Standard deviation, *CI* confidence interval

## Discussion

This trial studied two interventions delivered by HTs to reduce risky alcohol use: a 5-min BI accompanied by an informative leaflet, compared with the leaflet alone. Our results showed a reduction in the AUDIT risk status for the majority of the participants six months after receiving either the BI or the leaflet (i.e., 80% reduction in the BI group and 71% in the leaflet group) with no additional benefit attributable to the BI. Hence, this trial does not support the efficacy of a 5-min BI delivered by HTs when compared to a leaflet. However, there was a statistically significant reduction in the AUDIT score (secondary outcome) favoring the BI group, which might indicate some modest effect of the BI combined with the leaflet. However, we interpret this modest effect in the overall context of no efficacy shown by the primary outcome.

Our results depart from similar studies where health educators or lay providers have administered BIs with positive results. Possible explanations are that the providers´ training in those studies was more in-depth than in ours, and that the interventions were more intensive. Also, those providers had more experience delivering alcohol counselling. For example, in the United States, Bazargan-Hejazi et al. [26] conducted a non-randomized trial in emergency rooms where a 20-min BI was provided by health promotion advocates and found a reduction of 2.45 AUDIT points attributable to the BI. These community peer educators had previous experience in alcohol counselling and received more training than our HTs. In India, Nadkarni et al. [27] studied the effectiveness of an intervention provided by lay-counselors, finding that 36% of the participants in the intervention group had an AUDIT less than eight at follow-up; however, these participants had greater initial problematic alcohol use, the intervention was more intensive than ours, and the counsellors received a more in-depth training that enabled them to deliver a brief therapy for alcohol problems. In South Africa, Sorsdahl et al. [28] studied a BI administered by 'bachelor-level counselors' and found effectiveness associated with it; however, this intervention was also of higher intensity and encompassed both alcohol and other drug use.

It is plausible that a more intensive training and closer supervision of HTs could have yielded superior results of the BI over the leaflet. For example, Dhital et al. [29] designed a trial to inform policy makers in the United Kingdom on the effectiveness of a BI implemented by pharmaceutical chemists. These professionals had been identified as potential BI providers based on their extended community roles. However, this highly naturalistic study did not find effectiveness, and the researchers reported that little training and practice in the intervention might partly explain the lack of effectiveness.

Despite there being no difference in the AUDIT risk category among groups, we did observe a reduction of 0.89 AUDIT points attributable to the BI, when compared with the leaflet group at the six-month follow-up. Several clinical trials using the AUDIT as a primary outcome have shown reductions similar to those of the current study [27, 30–33]. Lane's study [30], in particular, where nurses provided a BI in an outpatient clinic, found a one-point greater reduction in the AUDIT score in the intervention group in comparison to the control group. However, the reduction we observed is small (Cohen's d = 0.21), thus limiting its clinical significance and making its public health impact uncertain.

More salient than any marginal BI effect was the reduction of 5 AUDIT points in the leaflet group. A definitive explanation of this effect is impossible to elucidate from the current study given that there was no control group; however, some known factors are plausibly involved. It is estimated, for example, that the evaluation of alcohol use produces a small reduction in reported use [34]. Also, a Hawthorne effect and simple regression to the mean are quite possible effects or explanations to the reduction [35]. Nonetheless, reductions in alcohol use of about 25% in the comparison group are commonly observed [36], as well as reductions close to 50%, as in our study, when active comparison groups are studied in PC [37, 38]. Another reasonable explanation for the favorable outcomes in the leaflet group is that a minimal intervention may produce an important reduction in the AUDIT risk category and score, as suggested by Kaner in a robust pragmatic trial [39].

### Study limitations

Comparing two active interventions instead of comparing the BI with a control group limits our results' interpretation in several ways. Mainly, our design with a leaflet group as a comparator impeded a direct estimation of the BI's net effect. Additionally, the inclusion of the leaflet in both groups reduced the experimental contrast among them. However, using this comparator was necessary because the leaflet is considered to be the minimal assistance provided through the national SBIRT program for people with risky alcohol use. In this regard, it was also a condition established by the ethics committees (i.e. not leaving people at risk without any support).

Also regarding the study´s design, both the providers and the participants were unblinded to allocation, which could have introduced performance bias among the HTs [40] or social desirability bias that influenced reporting of alcohol use by the participants [41].

Another limitation is a considerable risk of contamination between groups since the same HT provided the BI or leaflet according to the assignment. Nevertheless, the HTs were trained and supervised throughout the trial to minimize this risk. Additionally, a strength of our procedures is that the outcome adjudicators were blinded to the assignment, which supports the validity of our design. Another limitation of the current study is that the AUDIT was the only instrument used to recruit participants and measure the effects of the interventions. Although the limited measures collected in our study preclude further explanation of the findings, we believe that keeping the measurement to a minimum had the advantage of preserving the conditions in which the SBIRT program occurs and favored the recruitment of regular patients who may not have the time for lengthy procedures and interview questionnaires. Moreover, since additional measurements may generate a further decrease in alcohol use, as an unintended effect, we wanted to keep instruments to a minimum [42].

### Generalizability

The question about the efficacy of BIs delivered by health technicians is a pressing one in the Latin American context, where professional health workers are scarce. [43]. The present trial was conducted under conditions close to the real world so as to study the efficacy of this ongoing practice in Chilean PC. In this regard, our main conclusion is that the provision of a BI was not superior to the delivery of an informative leaflet for the reduction of risky alcohol consumption. These results have implications for the Chilean SBIRT program, particularly regarding how HTs are incorporated in the program. Mainly, they suggest that delivering an informative leaflet could be more efficient than delivering a 5-min BI.

Finally, from the current study design, it is not possible to explain the considerable risk reduction that occurred in the leaflet group (i.e., the decrease to low-risk category in 71% of the participants). Even though regression to the mean and contamination among groups are potential explanations, it is also likely that the leaflet intervention had some effect [39]. From a public health perspective, it is critical to elucidate if such a feasible minimal intervention could have an impact on the alcohol risk consumption at the population level. Future trials on this topic could be beneficial.

## Conclusions

The AUDIT-linked BI delivered by HTs was not associated with a greater reduction of risky alcohol consumption than an informative leaflet. Delivering a leaflet could be more efficient than a BI when provided by HTs; however, more research on the effectiveness of the leaflet is needed.

## Data Availability

The datasets generated and/or analysed during the current study are available in the Open Science Foundation repository, https://doi.org/10.17605/OSF.IO/AGBH9.

## Abbreviations

BI: Brief Intervention
MI: Motivational Interview
HT: Health Technician
AUDIT: Alcohol Use Disorders Identification Test
DALY: Disability Adjusted Life Year
SBIRT: Screening, Brief Intervention, and Referral to Treatment
PC: Primary Care
MOH: Ministry of Health
RCT: Randomized Controlled Trial
FONIS: National Health Research Fund
RA: Research Assistant
SD: Standard Deviation
CI: Confidence Interval
